# Quantum Cascade Laser-Based Vibrational Circular Dichroism Augmented by a Balanced Detection Scheme

**DOI:** 10.1021/acs.analchem.2c01269

**Published:** 2022-07-14

**Authors:** Daniel R. Hermann, Georg Ramer, Markus Kitzler-Zeiler, Bernhard Lendl

**Affiliations:** † Institute of Chemical Technologies and Analytics, TU Wien, Getreidemarkt 9/164-UPA, 1060 Vienna, Austria; ‡ Photonics Institute, TU Wien, Gußhausstrasse 27-29, 1040 Vienna, Austria

## Abstract

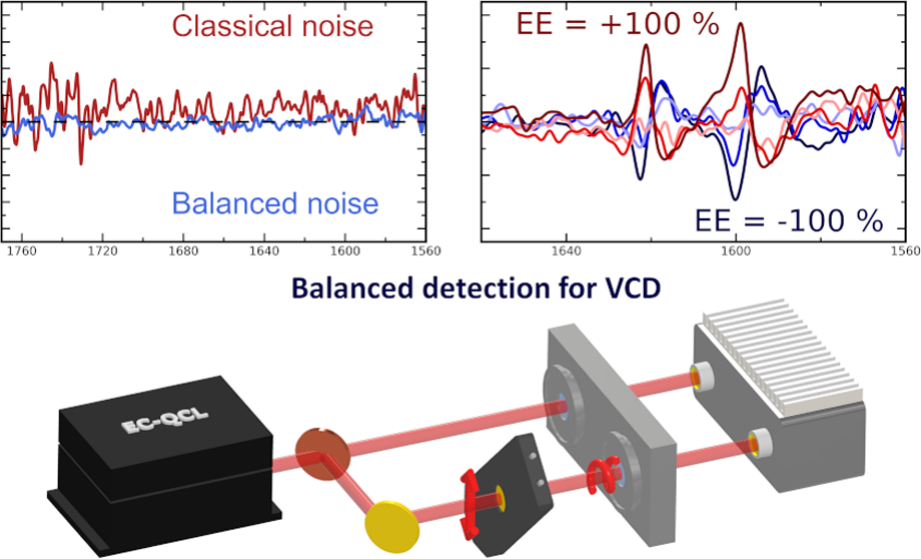

Vibrational circular dichroism (VCD) constitutes a powerful technique, enabling the determination of the absolute configuration of molecules without the need for specialized reagents. While delivering critical information, VCD signals commonly are several orders of magnitude weaker than classical absorbance signals, which so far necessitated long measurement times to achieve acceptable signal-to-noise ratios (SNRs) in VCD experiments. We present here an improved setup for the measurement of VCD in the range between 5.6 and 6.5 μm. Employing an external cavity quantum cascade laser (EC-QCL) as a high-power light source, we collected spectra with competitive noise levels in less than 5 min. The basis for this improvement was a balanced detection module combined with an optical path catered to VCD measurements. With the stabilization provided by the two-detector setup, noise originating from the laser source could be suppressed effectively. Noise level improvement up to a factor of 4 compared to the classical single detector EC-QCL-VCD could be reported. Compared to commercial Fourier transform infrared (FT-IR) instruments, the presented setup offers measurement time reductions of a factor of at least 6, with comparable noise levels. The applicability of the setup for qualitative and quantitative VCDs was proven. With the comparatively high temporal resolution provided, the monitoring of optically active processes will be possible in future applications.

Vibrational circular dichroism (VCD) is defined as the difference in absorbance for left and right circularly polarized lights in the infrared region (IR).^1^ VCD is sensitive to the absolute configuration of chiral molecules, as well as to the structure of larger biomolecules like proteins and nucleic acids.^1−3^ It is the logical extension of electronic circular dichroism (ECD or CD), which is the application of chiroptical spectroscopy in the ultraviolet and visible light ranges.^4,5^ While ECD has been routinely used for structural studies of proteins, it is limited to samples containing suitable chromophores.^1,5,6^ In contrast, IR, the spectral region in which VCD operates, is rich in band characteristics for different classes of organic molecules.^7^ Due to the significant increase of accessible molecules, VCD presents a more attractive tool for the study of chiral samples than ECD.^8,9^ Indeed, VCD has been used to straightforwardly determine the absolute configuration of small molecules and even monitor enantiomeric excess during reaction monitoring.^3,9,10^

While VCD offers obvious advantages due to its unique sensitivity to structural differences and a broad range of accessible samples, the collection of VCD spectra comes with significant challenges.^11−13^ VCD signals are 4–6 orders of magnitude weaker than classical absorbance, which necessitates low noise levels.^11,14^ Additionally, artifacts originating from birefringence can distort or overlap the already weak signals.^11,15^ The beginning of the VCD instrumentation lies in dispersive spectrometers, which used longer integration times by decreasing the scanning rate to obtain satisfactory signal-to-noise ratios. As with classical IR spectroscopy, Fourier transform infrared (FT-IR) spectrometers have replaced dispersive instruments for the measurement of VCD spectra.^11^ FT-IR instruments (and also dispersive instruments) can increase the signal-to-noise ratio by averaging increasing numbers of repeated spectral scans. This decreases the noise by √*n* (*n* is the number of averaged scans) and is well suited for modern FT-IR instruments capable of high scanning speeds.^14^ Since the noise levels necessary for obtaining usable VCD spectra are significantly lower than for classical absorbance measurements, the number of averaged scans and with it the measurement time increases substantially.^14^ Typical acquisition times for VCD range from ∼30 min to several hours, depending on the sample and solvent system.^16^ Especially challenging are samples in aqueous solutions since the high absorbance of water in the IR region significantly reduces the spectral throughput.^17,18^

Nearly all modern VCD instruments are based on FT-IR technology and employ thermal light sources like globars that for a long time were among the only broadly available light sources in the mid-IR region. One alternative is tunable mid-IR lasers. Here, quantum cascade lasers (QCLs), first introduced in 1994, are especially attractive for their high brilliance and intrinsically linearly polarized emission.^19^ Modern external cavity (EC)-QCLs offer tuning ranges of several hundreds of wavenumbers, large enough to observe multiple vibrational modes, and are available commercially for the whole mid-IR spectral range. Their high spectral output can be leveraged in highly absorbing samples, increasing the usable path length and the limit of detection.^20−22^

In 2008, Lüdeke et al. presented the first VCD spectra collected with an EC-QCL as a light source.^23^ The acquired spectra corresponded well with the classical FT-IR VCD spectra and proved the suitability of EC-QCLs for VCD experiments.^23^ However, despite the obvious useful properties of EC-QCLs, they also suffer from some disadvantages. Tunable QCLs feature a structured emission spectrum, with a maximum near the middle of the emitted spectral range and comparatively very weak spectral power at the spectral edges. Furthermore, intensity drifts, low-frequency noise, and, if the laser is operated in a pulsed mode, pulse-to-pulse fluctuations add additional noise to the system. Those noise sources are not present in classical thermal light sources.^22,24^

One approach for low noise laser spectroscopy is “balanced detection”: the laser beam is split into two beams of intensities as similar to each other as possible, which are directed onto two matched detectors. Only one light path contains the sample, while both channels carry the noise present in the laser. The signals collected from the detectors are then subtracted. This ensures that fluctuations present in the light source are canceled, as they are present in both channels. The signal of interest, on the other hand, is only present in one channel.^22,24^ This method was already successfully applied in mid-infrared EC-QCL absorbance measurements of proteins in solution, where a noise reduction by a factor of 20 compared to single detection schemes was demonstrated.^22^

In the current study, we extended the benefits of EC-QCL mid-IR spectroscopy with balanced detection to VCD measurements. A dedicated balanced detection scheme was implemented using two closely matched HgCdTe (MCT) detectors and a two-path transmission cell. Employing a photoelastic modulator (PEM), we introduced rotational sensitivity in one path of the balanced detection system, enabling the acquisition of EC-QCL-VCD spectra. The noise reduction provided by the application of a balanced detection scheme was evaluated, both in comparison with single detector schemes and with classical FT-IR VCD noise levels. Furthermore, the practical application of the balanced detection EC-QCL-VCD was investigated for qualitative studies as well as for enantiomeric excess studies with the enantiomeric pair R- and S-1,1′-bi-2-naphthol (BINOL).

## Experimental Section

### Reagents

CHCl_3_ (99.2%, stabilized with 0.6% ethanol), (R)-(+)-1,1′-bi-2-naphthol (R-BINOL, 99%), and (S)-(−)-1,1′-bi-2-naphthol (S-BINOL, 99%) were purchased from Sigma-Aldrich (St. Louis) and used without further purification. For sample solutions, the appropriate amount of pure sample was dissolved in CHCl_3_. Enantiomeric excess (EE) and samples with EE values of +100%, +60%, +20%, −20%, −60%, and −100% were prepared by mixing 100 mM R- and S-BINOL stock solutions. EE levels are calculated as
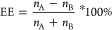
1with *n*_A_ and *n*_B_ referring to the molar concentration of components A and B, respectively (here: R- and S-BINOL).^9^

### Optical Setup

**Figure 1:**
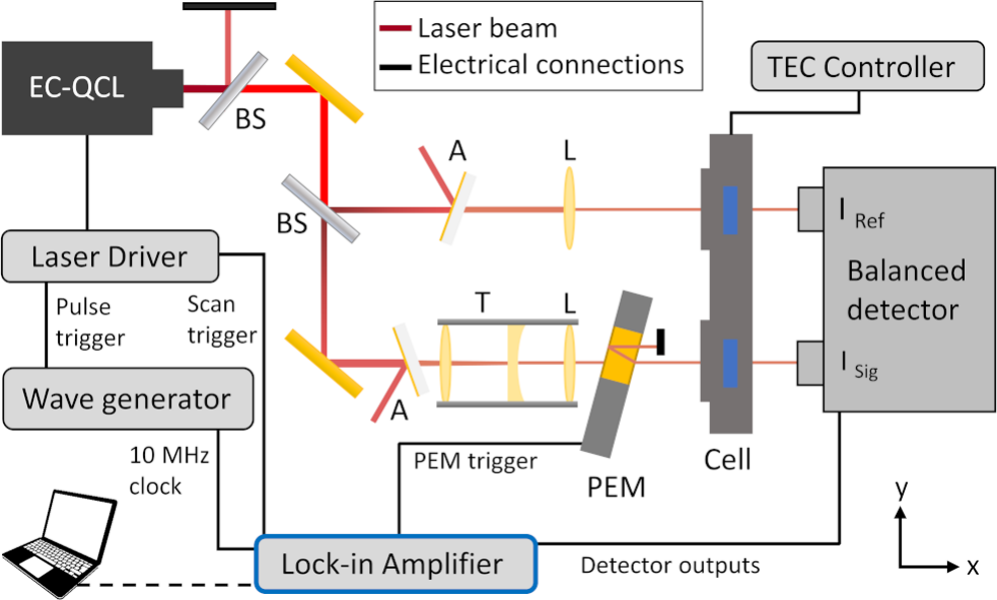
Optical setup used for the balanced VCD measurements. This setup encompasses an EC-QCL as a light source, beam splitters (BS), attenuators (A), a telescope system to reduce the beam diameter (T), focusing lenses (L), a photoelastic modulator (PEM), a double-path transmission cell (Cell), temperature stabilized by a thermoelectrical cooling (TEC) controller, and a balanced detector module.

Briefly, the high-sensitivity VCD EC-QCL spectroscopy setup introduces a polarization modulator into one arm of a balanced detection scheme (see Figure 1). An external cavity QCL (EC-QCL, Hedgehog, Daylight Solutions Inc., San Diego), tunable from 1780 to 1550 cm^–1^, was used as a light source. The EC-QCL emits a beam in the TEM_00_ spatial mode with linear polarization (vertical, nominally > 100:1) and a spot size of around 2.5 mm (1/e^2^ width). Operating with a current of 540 mA, a repetition rate of 1 MHz, and a pulse length of 200 ns, the laser reached a duty cycle of 20%. The laser head temperature was stabilized at 19 °C. Water cooling was used to remove excess heat.

A waveform generator (DG4102, RIGOL, Beijing, China) was used to trigger the laser. The internal clock of the waveform generator was synchronized to the lock-in amplifier used for signal recovery (see the Data Acquisition section). Spectra were collected in the “sweep mode”, whereby the EC-QCL tunes across its spectral range at a constant tuning speed without stopping. The sweep speed was set to 640 cm^–1^/s. Several attenuators were placed in the beam path before the sample to keep the intensity reaching the detector within the detector’s dynamic range. Attenuation was necessary, as decreasing the laser current results in a reduced spectral range of the EC-QCL.

The laser beam first passed a beam splitter (nominal 50/50), attenuating the beam by diverting half of the intensity onto a beam stop, and then was redirected by a gold mirror to another beam splitter (nominal 50/50). Here, the laser beam was divided into a reference and a sample beam (the reflected and the transmitted beams, respectively). Both the sample and the reference beams were diverted onto gold-sputtered BaF_2_ windows, wedged by 0.5° to prevent interference from reflections. (Sputtered windows were used instead of the commonly used metallic meshes, as the latter were found to strongly affect the beam profile.) The attenuated reference beam was focused by a ZnSe lens (*f* = 200 mm) through one cell of the double-path transmission cell onto the reference detector. All ZnSe lenses are sourced from Thorlabs (Newton, NJ) and AR-coated to a reflectivity below 1% between 4.5 and 7.5 μm. Reflective focusing was avoided, as reflections on metal surfaces show polarization-dependent bias and can introduce artifacts in the VCD spectra.^15,25,26^

The attenuated sample beam was directed into a cage system. First, the beam diameter was reduced by a Galilean telescope (*f* = 75 and −25.4 mm) to below 1 mm. Then, the beam was focused via a ZnSe lens (*f* = 200 mm, identical to the reference beam) onto the sample detector. After the cage system, the beam passed a 42 kHz ZnSe photoelastic modulator (Hinds instrument, Hillsboro) with a broad-band antireflection coating. The modulator was set to a quarter-wave retardation at 6 μm. For QCLs, the output polarization is oriented orthogonal to the gain medium layers, resulting in a vertical linear polarized laser beam, without the need for additional components like polarizers.^19^ The stress axis of the modulator was orientated at 45° relative to the laser polarization direction. To further reduce interference effects at the modulation frequency, the modulator was tilted by 15° around the vertical axis.^27^ In combination with the beam diameter reduction by the telescope, this allowed the blockage of the reflected beam. The now circular polarized laser light passed through the other cell in the double-path transmission cell.

The detector used in this setup is a balanced detection module with a manual gain regulation (Vigo System S.A., Poland). It is composed of two thermoelectrically cooled MCT detectors with a detectivity of 1.45 × 10^10^ cm Hz^1/2^/W (at 10.6 μm, 100 kHz). The detector elements and the corresponding electronics were chosen to ensure that both detectors have a similar response. This increases the efficiency of the common mode rejection in the balance detection scheme. Both detectors are equipped with a manual adjustable dark current compensation. The electrical gain of the reference channel can also be adjusted manually. Both sample and reference channel outputs are accessible via SMA connectors. Additionally, a third channel outputs the difference between the sample and reference voltage, determined via a differential amplifier in the detector. This additional channel is referred to as the balanced channel.

The custom-made double-path cell comprised two 160 μm long cells with CaF_2_ windows and was set to a temperature of 20 °C (±0.001 °C). The temperature was controlled by a thermoelectrical cooling controller (Meerstetter Engineering GmbH, Rubigen, Switzerland). The setup was encompassed in an acrylic glass housing and constantly purged with dry air to prevent the interference from water vapor during the spectra acquisition. For the spectra depicted in this work, approximately 400 μL of sample solution was manually injected and 700 spectra were averaged. This resulted in a total measurement time of 290 s (4 min 50 s). The classical absorbance spectra as well as the VCD spectra were baseline-corrected with the pure solvent measured under the same conditions.

### Data Acquisition

A lock-in amplifier (MFLI, Zurich Instruments, with F5M and MD extensions) was used to extract the phase-sensitive information from the detector signals. As mentioned, the laser pulsing scheme was controlled by a waveform generator, which was kept at a constant phase to the MFLI via a 10 MHz sync connection. Using the lock-in, the intensity *I*, which corresponds to the intensity of the laser after the sample, is recovered from the 1 MHz component of the detector output. The Δ*I*_L–R_ signal is the amplitude of the detector signal at the PEM modulation frequency *f*_PEM_, which carries the chiroptical information. The PEM outputs a rectangular pulse at *f*_PEM_. By feeding this signal to a phase-locked loop (PLL) of the lock-in amplifier, the phase and frequency of the PEM are determined and used to demodulate Δ*I*_L–R_.

It is important to keep in mind that in the balanced detection setup, not only Δ*I*_L–R_ but also *I* can be positive and negative; thus, for all signals, phase-sensitive detection is required. The correct phases were set when starting the experiment and were found to not require adjustment later. During the measurements, the *I* and Δ*I*_L–R_ components of the balanced output as well as the *I* of the reference detector output were collected. The general equation for calculating the VCD spectra is
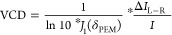
2with *J*_1_ being the first-order Bessel function and δ_PEM_ being the maximum retardation of the PEM.^7^ For the single channel configuration, the Δ*I*_L–R_ and *I* components are extracted from the sample channel detector signal and inserted into eq 2. For the balanced configuration, Δ*I*_L–R_ and *I* are extracted from the balanced channel output. At the beginning of a measurement day, the *I* component of the reference output is recorded. For each spectrum, the stored *I* is then added to the *I* component of the balanced output. The sum of balanced *I* and reference *I* is then used in eq 2 to calculate the VCD spectrum. The Δ*I*_L–R_ recorded from the balanced channel does not require a correction by a reference.

The sampling rate of the lock-in amplifier was set to 6696 Sa/s. For each wavenumber, 10 data points were collected, resulting in a total of 2300 data points per spectrum. All signals were demodulated using a fourth-order digital low pass filter (resulting in a filter roll-off of 24 dB/oct) and, in this case, a time constant of 745.8 μs (corresponding to 3 dB attenuation at 92.8 Hz). The time constant was chosen relative to the scanning frequency of the laser.

Before further evaluation, the spectra were low-pass-filtered using a finite impulse response (FIR) digital filter (Kaiser window, cutoff of 220 Hz). The wavenumber axis was calibrated using the positions of water vapor bands. By comparing the bandwidth of water vapor spectra collected with the setup to FT-IR spectra at different resolutions, a spectral resolution of 1.6 cm^–1^ was estimated.

### Partial Least-Squares Analysis

Multivariate data analysis was performed in Python 3 using the scikit-learn package,^28,29^ implemented based on in-house written scripts. No preprocessing was applied to the baseline-corrected enantiomeric excess VCD spectra, and a leave-one-out cross-validation method was applied. A partial least-squares (PLS) regression was constructed, with the enantiomeric excess levels and the baseline-corrected VCD spectra being used as response and independent variables, respectively. Since the data set was quite small (*n* = 18, 6 levels in 3 replicates), this method was appropriate. The chosen spectral range was optimized from 1653 to 1561 cm^–1^ based on the root-mean-square error of the cross-validation (RMSECV). The model was built with two latent variables chosen based on the optimal RMSECV while avoiding overfitting. Besides cross-validation, external validation was performed with five samples (EE levels: −100%, −60%, −20%, 0%, +20%) prepared with different stock solutions and measured on a different day.

## Results and Discussion

### Applicability for VCD

**Figure 2:**
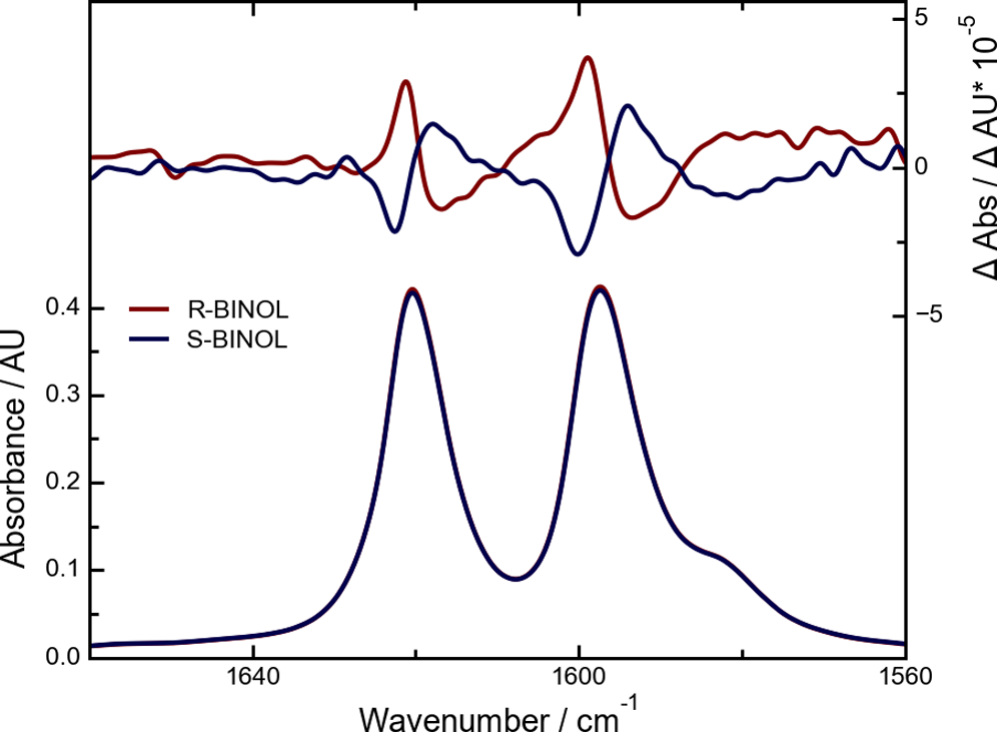
Absorbance (left axis) and VCD (right axis) of R-BINOL (blue) and S-BINOL (red) in CHCl_3_. The samples were measured at a concentration of 0.1 M with a path length of 160 μm. 700 scans were averaged, corresponding to a 290 s integration time.

In Figure 2, the absorbance and VCD spectra of 100 mM R- and S-BINOL in CHCl_3_ are shown. The absorbance and VCD spectra were baseline-corrected with the pure solvent. As expected, no difference between the absorbance spectra of the two enantiomers is observed, whereas their VCD spectra show a clear mirror image relation. The position and orientation of the bands in the VCD spectrum match those from the literature recorded using conventional FT-IR VCD instrumentation well.^30,31^

### Balanced Detection for VCD Noise Reduction

**Figure 3:**
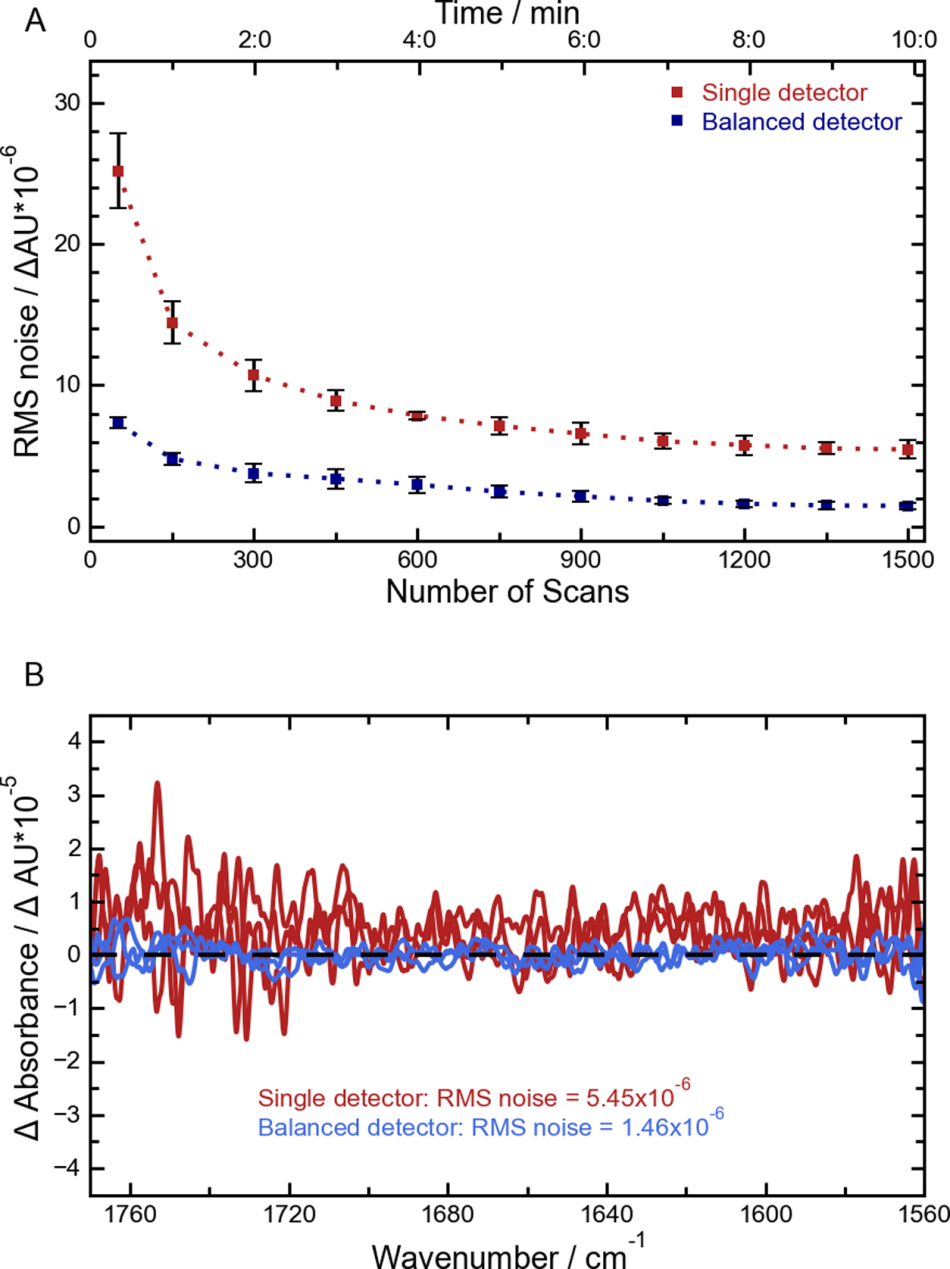
(A) RMS noise level in the single detector configuration and the balanced detector configuration. The RMS noise level (average of five measurements) of the VCD spectra for each configuration is plotted as a function of integration time and number of scans used for averaging. The error bars correspond to the standard deviation between the measurements. (B) Typical noise spectra for VCD spectra of CHCl_3_ obtained after averaging 1500 spectra for single or balanced detector configuration. The RMS noise value for each configuration is also shown.

The noise in single detector and balanced detection was evaluated as the difference between two subsequent VCD spectra of the solvent (CHCl_3_). Ideally, the result should be a zero line.^17,21^ Examples of these noise spectra are shown in Figure 3B. The potential for improving noise levels through the averaging of scans was evaluated for both single detector and balanced detection. Up to 1500 scans were averaged before calculating the RMS noise levels between 1600 and 1730 cm^–1^. In Figure 3A, the average RMS value of five replicates is plotted against the number of averaged scans.

The performance of the balanced detection scheme is better than that of the standard single detector method, with a noise reduction by a factor of around 3.7. As this reduction shows promise, the improvement is not as large as we expected from comparable works with classical IR absorbance.^22^ A probable explanation for smaller improvement is the normalization inherent to VCD spectra. To obtain raw VCD spectra, the signal collected at the PEM frequency is divided by the one collected at the laser frequency. Through this process, parts of the pulse-to-pulse fluctuations are compensated regardless of the detector configuration. Nevertheless, the data presented still show improvement in the noise levels by the application of balanced detection as well as an increased repeatability of the spectra. This is shown by the reduced deviations (error bars) between the replicates for the balanced detection scheme when compared to those for the single detector scheme.

**Figure 4:**
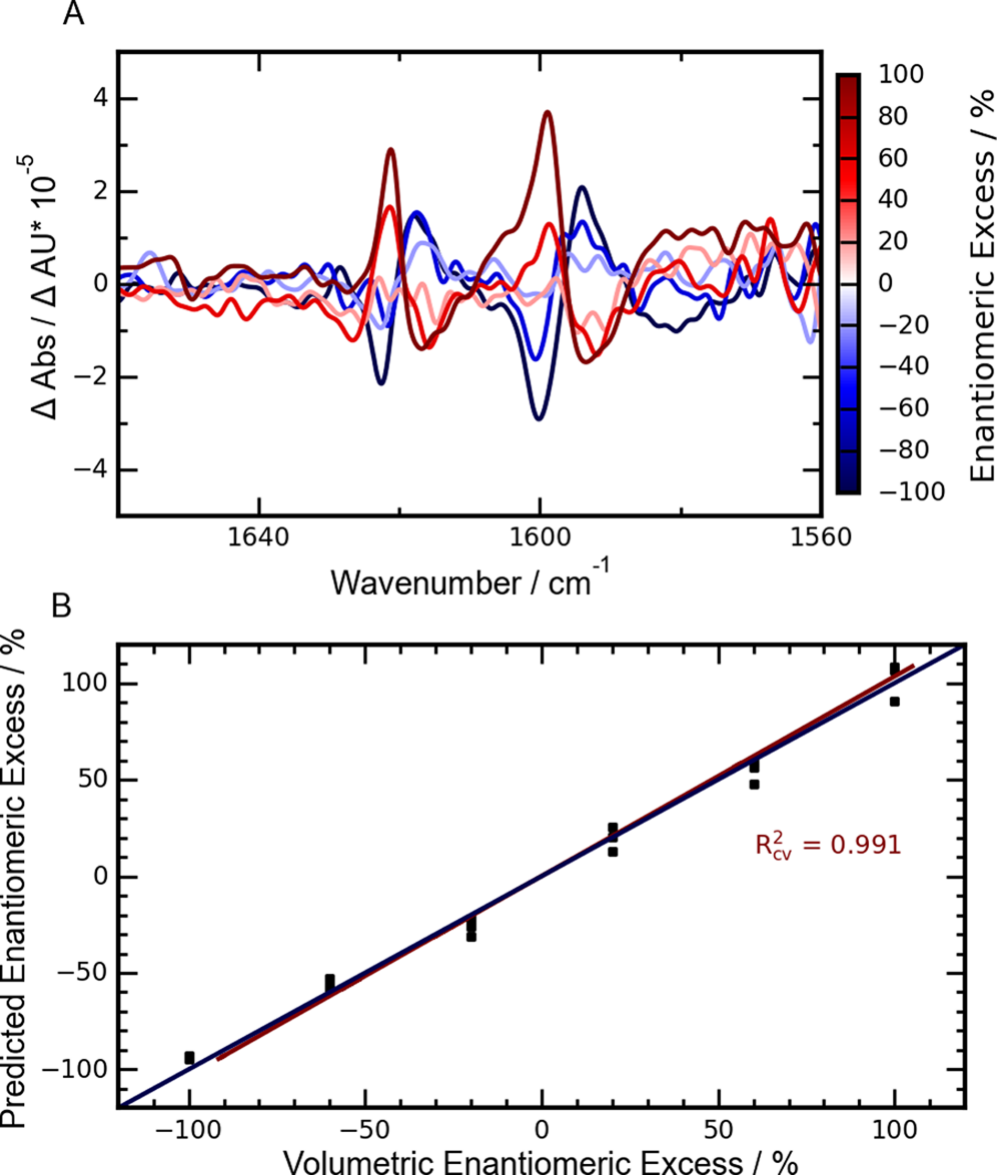
(A) VCD spectra obtained by measuring different levels of enantiomeric excess. For the corresponding enantiomeric excess level, the reader is referred to the depicted color bar. The concentration of BINOL was 100 mM in a cell with a path length of 160 μm, and 700 spectra were averaged. (B) Linear correlation of the predicted enantiomeric excess values (cross-validated) and the volumetric enantiomeric excess of the prepared samples. The obtained fit is plotted in red, with the corresponding *R*^2^ being depicted beside the graph. The blue line indicates a perfect correlation.

The superior performance of the balanced detection scheme also becomes apparent in the spectra shown in Figure 3B. Here, typical VCD noise spectra for the single and the balanced detection setup are overlaid. The baseline produced by the balanced detection stays around zero for the whole spectral range, with no clear peaks visible. In comparison, the baseline obtained from the single detector scheme deviates considerably from the zero line. When comparing the baselines to the peaks depicted in Figures 2 and 4A, this improvement can be contextualized. Especially for lower EE values, the peaks are in the range of the noise floor of the single detector baseline. This would hinder a correct interpretation of the spectra, necessitating longer measurement times to decrease the noise to sufficient levels.

Biological samples, due to their inherent chirality and structural orientation, present themselves as obvious analytes for VCD studies. However, proteins, for example, seldom exhibit VCD signals more intense than 2 × 10^–5^.^18,32^ For these samples, the reduced noise of the balanced detection setup is an obvious advantage. Protein spectra typically take several hours to collect with classical FT-IR spectrometers. A single detector scheme would need a significantly longer measurement time than the balanced detection setup and offer less improvement compared to FT-IR spectra.

In addition to the graphs in Figure 3, the overall noise performance is summarized in Table 1. All shown values are calculated between 1730 and 1600 cm^–1^ for spectra collected by averaging 1500 scans (10 min measurements). The values for the FT-IR performance are taken from the literature. As already mentioned, the RMS noise values for the balanced detection are around 3.7 times lower than for single detector setup, being 1.45 × 10^–6^ compared to 5.45 × 10^–6^. The available FT-IR data are collected with 3 times longer averaging times and reach RMS noise levels of 2.8 × 10^–6^.^33^ For balanced operation, the EC-QCL-based setup reached a noise level nearly 2 times better in a third of the measurement time. Besides the RMS noise, the noise height is also a relevant noise parameter for VCD spectra. The relevant chapter ⟨1782⟩ of the U.S. pharmacopeia defines it as the maximum deviation from the zero line over the spectral range of interest.^34^ This value was also calculated and is shown in Table 1. The noise height for the balanced detection system is almost 4 times lower than for a single detector system, with 3.83 × 10^–6^ and 15.2 × 10^–6^, respectively. The available FT-IR data were collected after 60 min of averaging and deviated from zero by 5 × 10^–5^, 13 times higher than the deviation achieved for the balanced detection setup.

**Table 1:** Noise Characteristics for the Single Detector Scheme, the Balanced Detection Scheme, and Values for FT-IR Spectrometer from the Literature

	RMS noise[10-6 ΔAU]	time [min]	noise height [10-6 ΔAU]	time [min]
single	5.45	10	15.2	10
balanced	1.46	10	3.83	10
FT-IR^33,34^	2.8	30	50	60

Besides lower noise levels and shorter measurement times, our EC-QCL-based setup also offers a higher spectral resolution. Typical FT-IR VCD spectra are collected at resolutions between 4 and 8 cm^–1^, while our data were presented at a resolution of 1.6 cm^–1^.

### Enantiomeric Excess Studies

In addition to studying the spectrum of enantiomers, VCD also allows the quantification of enantiomeric excess. This application is more challenging as the bands in VCD spectra of mixed enantiomers are lower than in enantiopure samples. Solutions with different levels of enantiomeric excess were prepared and measured in triplicate. The EE levels were varied from +100 to −100 in steps of 40 %. The resulting spectra are shown in Figure 4A. With decreasing enantiomeric excess levels, the VCD couplets centered at 1619 and 1596 cm^–1^, respectively, changed their orientation. The negative couplets (negative VCD intensity at the higher wavenumber, positive intensity at lower wavenumber) characteristic of R-BINOL decrease in intensity and evolve into the positive couplets expected from S-BINOL. The intensity and orientation of the couplet centered at 1596 cm^–1^ were used to describe the enantiomeric excess in a univariate linear model. The resulting correlation line can be found in the Supporting Information (see Figure S4). The *r*^2^ of 0.997 indicates a high correlation, and the *p*-value of the slope (*p* = 3.3 × 10^–6^) attests the significance of the correlation. The limit of detection (LOD) for the EE was calculated according to
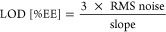
3where RMS noise stands for the root-mean-square (RMS) noise, and slope stands for the slope of the calibration curve.^22^ The obtained value was 10.5 % EE. Taking the used total (R- + S-BINOL) concentration of 100 mM into account, this corresponds to a concentration difference of 5.25 mM between the enantiomers.

The use of a multivariate method such as partial least-squares regression (PLSr) could help to further improve this value, as these methods are less susceptible to noise over a few wavenumbers. To test this, a PLSr model was constructed based on the spectra shown in Figure 4A. The predictions obtained from this model can be seen in Figure 4B. The predicted (cross-validated) and actual volumetric enantiomeric excess levels show a high correlation with an *R*^2^ of 0.991. The *R*^2^ of the calibration model was 0.996, and the root-mean-square error (RMSE) was 4.31 % EE. Further statistical parameters can be seen in Table 2.

**Table 2:** Statistical Parameters for the PLS Model Constructed Based on the Enantiomeric Excess Study

	*R*^2^	RMSE [%]
calibration^a^	0.996	4.31
cross-validation^b^	0.991	6.64
prediction^c^	0.964	8.16

a Full data set (6 levels, 3 replicates = 18 data points).

b Leave-one-out cross-validation.

c Five unrelated samples (EE: −100 to +20). RMSE: root-mean-square error.

To validate the performance of the model, a leave-one-out cross-validation was performed. Due to the small sample size, this approach is appropriate. The cross-validation showed an *R*^2^ of 0.991 and a slightly higher RMSE of 6.64 % EE than for the calibration. Since the calculation of the LOD for the multivariate method is not as straightforward as for univariate models, the RMSE of the cross-validation was taken as a measure of accuracy.^35^ This value corresponds to a concentration difference of 3.32 mM in our case. To further evaluate the applicability of the model, external validation was performed with five samples prepared and measured on a different day. The obtained *R*^2^ of 0.964 indicates a still high correlation, and an RMSE of 8.16 % is comparable to the RMSE of the cross-validation. This indicates a robust model, without the presence of overfitting.

Enantiomeric excess prediction by VCD was already studied, with great success on FT-IR instruments. While these studies achieved better RMSECV than the one we performed in these experiments, this comparison needs to be contextualized^3,9,36^ Since the studies were performed with substantially different concentration ranges, the RMSECVs were normalized to the molar concentration. The resulting detectable molar differences attested to our setup a performance comparable to or better than most published results, with measurement times reduced by a factor of 4.^3,9^ Only one study achieved a better sensitivity, which was enabled by a measurement time longer by a factor of 120.^36^ Based on these data, the advantages provided by balanced detection EC-QCL-VCD enabled enantiomeric excess studies with comparatively low concentrations (100 mM) and low VCD signals while still providing a high temporal resolution. This can extend the applicability of VCD to samples where only low concentrations are accessible and which are characterized by low VCD signals without losing temporal resolution. The accuracy and robustness of the model could be improved by measuring additional sample points, applying longer measurement times, and evaluating different concentration ranges. However, this is beyond the scope of this study.

## Conclusions

We presented an improved method to measure EC-QCL-based VCD spectra. The combination of an EC-QCL and a balanced detection module used in the study enables the leveraging of the high brilliance of EC-QCLs without introducing additional noise into the spectra. Relevant noise parameters were improved by up to a factor of 4 compared to single detector measurements. The applicability for VCD measurements was examined with qualitative and quantitative experiments using R- and S-BINOL as model substances. Enantiomeric excess studies were possible with measurement times below 5 min for samples with low molar concentrations. The noise levels are significantly lower than for commercial FT-IR instruments even at acquisition times shorter by a factor of up to 6.

Of course, this comparison needs to be contextualized by the strengths and weaknesses of FT-IR and EC-QCLs, respectively. FT-IR instruments offer a broad coverage (up to several thousand wavenumbers) with a constant noise floor over this range while being restricted in terms of sensitivity by the relatively weak light source. EC-QCLs, on the other hand, provide a comparatively limited spectral coverage (several hundred wavenumbers) and are characterized by a higher noise level at the edges of their spectral emission but can leverage their high intensity to minimize limits of detections even with strongly absorbing solvents. Due to these differences, the perfect technique depends mostly on the envisioned application. FT-VCD has an edge when dealing with the absolute configuration determination or complex sample mixtures with multiple bands over a broad spectral area. EC-QCL-VCD, on the other hand, can excel in applications like EE determination or also with biomolecules like proteins in an aqueous solution. For these applications, a smaller spectral area is perfectly sufficient, and the higher sensitivity provided by the EC-QCL can prove to be advantageous here.

Employing a dedicated balanced detection scheme for VCD measurements enables the collection of low noise spectra with a high temporal and spectral resolution when compared to state-of-the-art FT-IR VCD instruments. This allows a comprehensive monitoring of processes where the chirality of the sample changes over time, like folding mechanisms of biological molecules. Indeed, our previous work on EC-QCL suggests that the high spectral power density of the laser will provide further gains in sensitivity over FT-IR when analyzing samples in aqueous solutions.^22^

## Supporting Information Available

Noise height against measurement times, detector signals at the laser frequency for the reference, the sample and the balanced channel, detector signals at the PEM frequency for the sample and the balanced channel, and univariate calibration line for the enantiomeric excess study (PDF)
