# Supplementary material for: Quantum Cascade Laser-Based Vibrational Circular Dichroism Augmented by a Balanced Detection Scheme
Source: Anal Chem. 2022 Jul 14;94(29):10384–90. doi: 10.1021/acs.analchem.2c01269 (PMC9330291; doi:10.1021/acs.analchem.2c01269)
Supplement: Supplementary file 1 — ac2c01269_si_001.pdf [file ac2c01269_si_001.pdf]

Supporting Information

Quantum Cascade laser based Vibrational Circular Dichroism augmented by a balanced detection scheme

Daniel R. Hermann, † Georg Ramer, † Markus Kitzler-Zeiler‡ and Bernhard Lendl†, *

†Institute of Chemical Technologies and Analytics, TU Wien, Getreidemarkt 9/164-UPA, 1060, Vienna, Austria.
‡Photonics Institute, TU Wien, Gußhausstrasse 27-29, 1040 Vienna, Austria.

Description of Contents

Figure S1. Deviation from zero against the measurement time S-2

Figure S2. Noise between subsequent blocks of sample spectra S-2

Figure S3. (A) Detector signal for the Reference, Balanced and Sample Channel, demodulated at laser frequency S-3

Figure S3. (B) Detector signal for the Reference, Balanced and Sample Channel, demodulated at PEM frequency S-3

Figure S4. Univariate Calibration line obtained for the enantiomeric excess studies. S-3

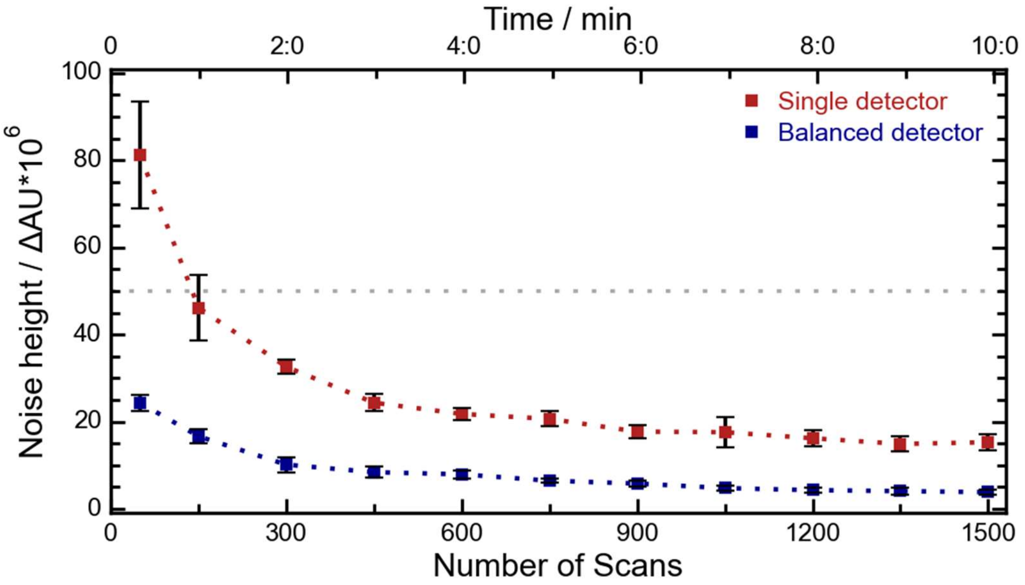

Figure S1. Plot of the maximum deviation from zero against the number of averaged spectra and the measurement time. The level published for FT-IR instruments at 60 minutes averaging is indicated by the grey dotted line. The error bars indicate the standard deviation of 5 replicate measurements.

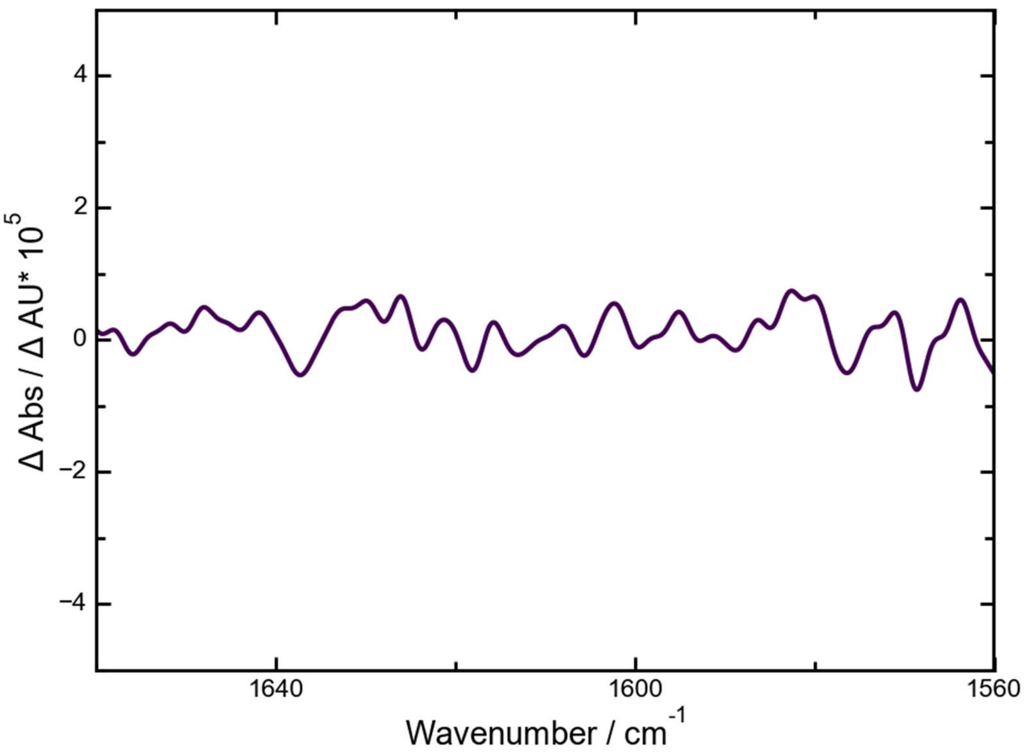

Figure S2. Noise level between two blocks of 700 scans for a sample of 100 % EE. Shown to compare the setup performance to FT-IR instruments, as the difference between subsequent scans of a sample is often used as an indicator for the noise level. The scaling of the plot is comparable to Figure 4 (A)

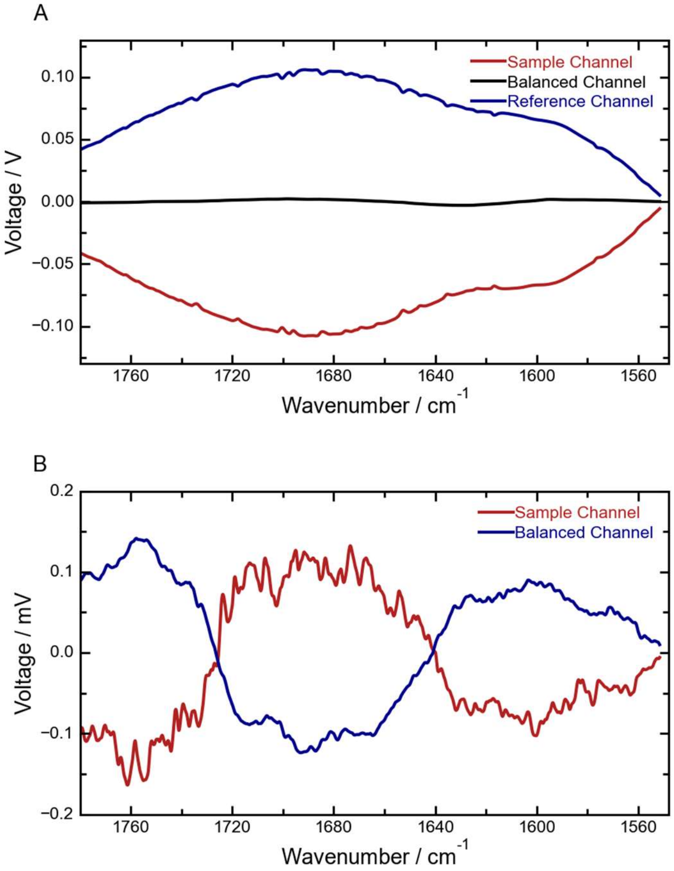

Figure S3. (A) Detector signal for the Sample, the Reference detector and the output from the differential amplifier, demodulated at the laser frequency. (B) Detector signals collected for the Sample and the balanced channel output, demodulated at the PEM frequency. Due to the electronical configuration of the balanced and the signal output, the two signals have opposite signs.

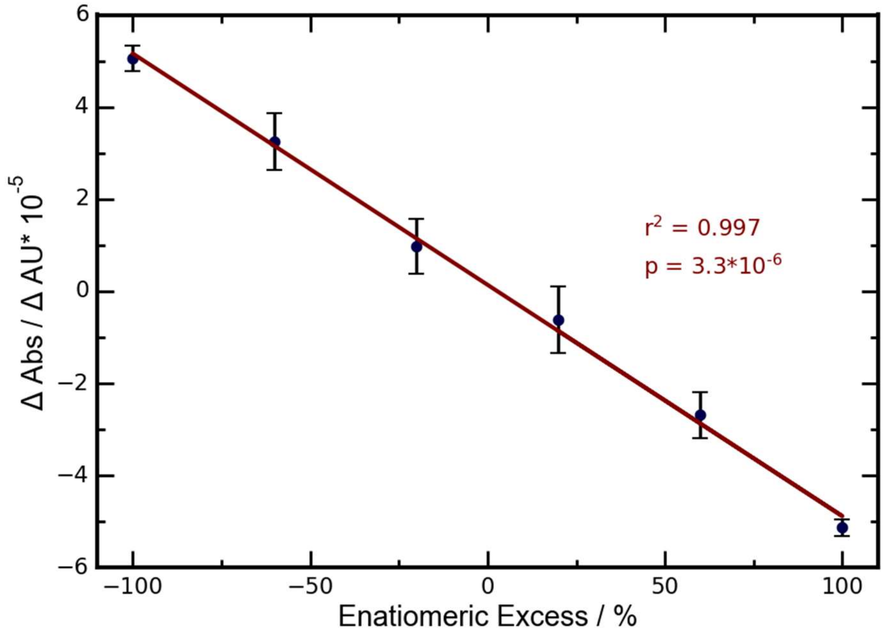

Figure S4. Calibration line, obtained by plotting the signal amplitude of the couplet centered at 1596 cm-1 against the enantiomeric excess. The error bars signify the standard deviations of 3 replicate measurements, and the r2 of the calibration line and the p-value of the slope are depicted besides the graph.
